# Terabase Metagenome Sequencing of Grassland Soil Microbiomes

**DOI:** 10.1128/MRA.00718-20

**Published:** 2020-08-06

**Authors:** William C. Nelson, Lindsey N. Anderson, Ruonan Wu, Jason E. McDermott, Sheryl L. Bell, Ari Jumpponen, Sarah J. Fansler, Kimberly J. Tyrrell, Yuliya Farris, Kirsten S. Hofmockel, Janet K. Jansson

**Affiliations:** a Earth and Biological Sciences Directorate, Pacific Northwest National Laboratory, Richland, Washington, USA; b Department of Ecology, Evolution, and Organismal Biology, Iowa State University, Ames, Iowa, USA; c Division of Biology, Kansas State University, Manhattan, Kansas, USA; Georgia Institute of Technology

## Abstract

To enable an in-depth survey of the metabolic potential of complex soil microbiomes, we performed ultra-deep metagenome sequencing, collecting >1 Tb of sequence data from three grassland soils representing different precipitation regimes.

## ANNOUNCEMENT

As part of the Pacific Northwest National Laboratory (PNNL) Science Focus Area program (1, 2), we are investigating the impact of environmental change on microbial community function in grassland soils. Three grassland soils, representing different moisture regimes, were selected for ultra-deep metagenome sequencing, resulting in >1 Tb of sequence data per location. This data set serves as a resource for deep analysis of soil microbiome composition and metabolic potential.

Soils were collected from three grassland field site locations. Arid regime soil (irrigated agriculture), characterized as a coarse silty loam, was collected from the Washington State University Irrigated Agriculture Research and Extension Center (IAREC) (46.25N, 119.73W). Intermediate precipitation regime soil (rain-fed and irrigated agriculture), characterized as a fine clay loam, was collected from the Konza Prairie Biological Station (KPBS) (39.10N, 96.61W) (3, 4). Frequent precipitation regime soil (rain-fed and tile-drained agriculture), characterized as a fine silty clay loam, was collected from the Iowa State University Comparison of Biofuel Systems (COBS) (41.92N, 93.75W) (5).

Surface soil samples (2 cm by 0 to 20 cm) were collected from three randomly selected field site block locations using a push corer (3 subsamples per block, 3 replicates per subsample). Replicate subsamples were sieved together, resulting in 9 independent samples per site. Samples were flash frozen and stored at −80°C until further processing.

DNA was extracted from 3 × 0.25 g soil for each of the 9 field samples per site using the PowerSoil DNA extraction kit (Qiagen), with bead beating, and quantified. The extracted DNA samples from each site were combined to generate a pooled sample from each location (IAREC, COBS, and KPBS) for sequencing. Metagenomic libraries were prepared using the TruSeq PCR-free kit (Illumina) and a starting material of 1 μg DNA from the pooled DNA. Sequencing was performed on an Illumina HiSeq X system at Fulgent Genetics (Los Angeles, CA), generating 150-nucleotide paired-end reads to a final effort of at least 1 Tb of sequence per site (Table 1). BBDuk (BBTools package v38.38) (6) was used to trim adapter sequences from raw reads (adapters_no_transposase database), to perform quality filtering (parameters: int, ow; k, 27; hdist, 1; qtrim, f; minlen, 35), and to remove contaminants (sequencing_artifacts and phix174_ill reference database). Assembly was performed using the metaHipMer assembler (see MIMS metadata files for the specific developmental version used for each site) with kmer lengths of 21, 31, 55, and 71 (7) on the NERSC Cori platform (https://docs.nersc.gov/systems/cori). Scaffolds <2,500 bp long were omitted from further analysis. Quality-screened reads were mapped to scaffolds using the Burrows-Wheeler Aligner (v0.7.12) (8), and depth of coverage was determined across each scaffold using SAMtools (v1.9) (9).

**TABLE 1:** Metagenome statistics for grassland soil microbiomes

Site	Total no. of reads	No. of quality bases (Tb)	Total no. of scaffolds	*N*_50_ (bp)	No. of scaffolds ≥2,500 bp	Total length of scaffolds ≥2,500 bp (bp)	No. of predicted proteins	PNNL DataHub accession no.
IAREC	7,536,393,634	1.123	83,651,096	1,404	241,472	989,234,018	1,255,684	WA-TmG.1.0
KPBS	7,343,389,182	1.088	68,100,771	1,111	304,736	1,283,171,244	1,388,888	KS-TmG.1.0
COBS	7,723,367,404	1.152	77,470,427	1,194	289,845	1,152,748,070	1,255,684	IA-TmG.1.0

Prodigal (v2.6.3) (10) was used to predict coding regions. Predicted protein sequences were searched using hmmsearch (v.3.1b2) (11) against the eggNOG (v4.5) (12), Pfam (v32.0) (13), and Nucleo-Cytoplasmic Virus Orthologous Group (NCVOG) (release date, 9 June 2014) (14) databases. Annotation assignments were given based on best bit scores (E-value cutoff, 1.0e−05).

These metagenomes are intended as a resource for the scientific community and should facilitate understanding of the highly diverse and complex metabolic potential that is encoded in soil microbial genomes.

### Data availability.

Metagenomic sequence data have been deposited in the PNNL DataHub repository and are available for download under project doi numbers WA-TmG.1.0, KS-TmG.1.0, and IA-TmG.1.0. The versions described in this paper are the first versions. Packages contain raw reads, assemblies, functional annotations, field site plot maps, MIMS.me.soil.5.0 metadata information, and package “read me” files.
